# Impact of Conventional and Atypical MAPKs on the Development of Metabolic Diseases

**DOI:** 10.3390/biom10091256

**Published:** 2020-08-29

**Authors:** Toufic Kassouf, Grzegorz Sumara

**Affiliations:** Dioscuri Centre for Metabolic Diseases, Nencki Institute of Experimental Biology, Polish Academy of Sciences, 3 Pasteur Street, 02-093 Warsaw, Poland; t.kassouf@nencki.edu.pl

**Keywords:** obesity, diabetes, MAPKs, ERK1/2, JNKs, p38, ERK5, ERK3, ERK7, NLK

## Abstract

The family of mitogen-activated protein kinases (MAPKs) consists of fourteen members and has been implicated in regulation of virtually all cellular processes. MAPKs are divided into two groups, conventional and atypical MAPKs. Conventional MAPKs are further classified into four sub-families: extracellular signal-regulated kinases 1/2 (ERK1/2), c-Jun N-terminal kinase (JNK1, 2 and 3), p38 (α, β, γ, δ), and extracellular signal-regulated kinase 5 (ERK5). Four kinases, extracellular signal-regulated kinase 3, 4, and 7 (ERK3, 4 and 7) as well as Nemo-like kinase (NLK) build a group of atypical MAPKs, which are activated by different upstream mechanisms than conventional MAPKs. Early studies identified JNK1/2 and ERK1/2 as well as p38α as a central mediators of inflammation-evoked insulin resistance. These kinases have been also implicated in the development of obesity and diabetes. Recently, other members of conventional MAPKs emerged as important mediators of liver, skeletal muscle, adipose tissue, and pancreatic β-cell metabolism. Moreover, latest studies indicate that atypical members of MAPK family play a central role in the regulation of adipose tissue function. In this review, we summarize early studies on conventional MAPKs as well as recent findings implicating previously ignored members of the MAPK family. Finally, we discuss the therapeutic potential of drugs targeting specific members of the MAPK family.

## 1. Introduction

Mitogen-activated protein kinases (MAPKs) are protein Ser/Thr kinases that convert a variety of extracellular signals into a wide range of cellular responses. In mammals, 14 MAPKs, including both conventional and atypical MAPKs, have been characterized and divided into seven subfamilies [1,2]. Conventional MAPKs comprise four subfamilies of MAPKs: the extracellular signal-regulated kinases 1/2 (ERK1/2), c-Jun amino (N)-terminal kinases 1/2/3 (JNK1/2/3), p38 isoforms (p38α, β, γ, and δ), and ERK5. Activation of each group of conventional MAPKs depends on a set of two upstream, evolutionarily conserved, sequentially acting kinases [2]. Each MAPK is activated by dual phosphorylation of a tripeptide motif (Thr-X-Tyr) located in the activation loop (T-loop). This phosphorylation is mediated by a MAPK kinase (MAPKK) that is activated by phosphorylation by an upstream MAPKK kinase (MAPKKK) [2]. Atypical, those having nonconforming particularities, MAPKs on the other hand comprise ERK3/4, ERK7, and Nemo-like kinase (NLK). In contrast to conventional MAPKs, they are not organized into classical three-tiered kinase cascades, and less is known about the exact molecular mechanisms involved in their activation [1,2].

MAPK signaling pathways regulate a variety of cellular activities including proliferation, differentiation, survival, and death [2]. Consistent with their critical roles in key cellular activities, deregulation of these pathways has been implicated in the pathogenesis of many human diseases including Alzheimer’s disease, Parkinson’s disease, amyotrophic lateral sclerosis, and various types of cancers [3]. MAPKs are also required for an array of metabolic events, and consequently inappropriate MAPK signaling contributes to the development of several metabolic diseases including obesity, type 2 diabetes (T2D) as well as non-alcoholic fatty liver disease (NAFLD) and atherosclerosis [4,5,6]. 

In this review, we discuss the relationship between conventional and atypical MAPK-signaling mechanisms that underlay metabolism and metabolic disorders.

## 2. The Conventional MAPKs

### 2.1. ERK1/2 Kinases

ERK1 and ERK2, also known as MAPK3 and MAPK1 respectively, are evolutionarily conserved kinases encoded by two separate genes and expressed to different extent in all tissues, with particularly high levels in the brain, skeletal muscle, thymus, and heart [7]. ERK1/2 are activated by growth factors, ligands for heterotrimeric G protein-coupled receptors (GPCRs), cytokines, osmotic stress, microtubule disorganization, and in response to insulin [2]. Their main initiating kinases (MAPKKK) are members of the RAF family, which include serine/threonine-protein kinase A-raf (ARAF), BRAF, and CRAF, and are often activated as a result of their interaction with active GTP-bound RAS proteins. Once activated, RAF kinases phosphorylate and activate components of the MAPKK module, such as MEK1 and MEK2, which in turn activate two MAPKs, ERK1, and ERK2, through phosphorylation of both tyrosine and threonine residues present in a conserved tripeptide motif (Thr-Glu-Tyr) within their activation loop [2]. Through the phosphorylation of their targets, ERK1 and ERK2 are involved in a wide variety of cellular processes including proliferation, inflammation, and cellular metabolism (reviewed in [2,8]). Upon activation, ERK1/2 phosphorylate a large number of substrates localized in nucleus and cytosol (Table 1). In the cytosol, ribosomal S6 kinase (RSK) family members, which are known to inactivate apoptotic proteins and to promote cell proliferation, represent one of the major substrates for ERK1/2 [9]. MAPK-interacting kinases 1/2 (MNK1/2) and Mitogen- and stress-activated kinase 1/2 (MSK1/2) are also important downstream targets of the ERK1/2 [10,11]. MNK1/2 control translation of mRNA [11]. MSKs phosphorylate multiple substrates, including cAMP-responsive element-binding protein (CREB) and Histone H3, and their major role is the regulation of specific subsets of immediate early genes (IEG) [10]. In the nucleus, ERK1/2 phosphorylate and activate the transcription factor ETS domain-containing protein Elk-1 (Elk-1), which mediates the transcription of c-Fos [12]. ERK1/2 are also known to stabilize c-Fos and to prevent its degradation, allowing c-Fos to associate with c-Jun and form transcriptionally active complexes. c-Jun/c-Fos dimer promote expression of cyclin D1, a protein that interacts with cyclin-dependent kinases (CDKs) and permits G1/S transition and cell cycle progression [13,14]. ERK1/2 are also known to mediate cell motility by phosphorylating actin-binding proteins implicated in cytoskeletal remodeling [15]. They are also involved in actin polymerization via their interaction with different actin regulatory proteins [16]. Additionally, ERK1/2 are implicated in the development of multiple types of cancers (reviewed in [17,18]). Consistent with their critical roles in tumorigenesis, targeting these enzymes present nowadays an outstanding interest for different therapeutic purposes [18].

In vivo ERK1 or ERK2 abrogation leads to different phenotypes, demonstrating different roles for the two isoforms. Disruption of the *Erk2* locus results in embryonic lethality early in mouse development after the implantation phase. On the contrary, mice deficient of *Erk1* in the whole body are viable and fertile [19]. Interestingly, transgenic expression of ERK1 fully rescues the embryonic and placental developmental defects resulting from the loss of ERK2, suggesting functional redundancy during development [20]. Most importantly, ERK1/2 pathways are central in regulation of metabolism in the multiple organs.

#### 2.1.1. The Role of ERK1/2 in the Liver

The liver plays a major role in maintaining glucose homeostasis by releasing glucose into the circulation via glycogenolysis and gluconeogenesis during fasting [21]. Moreover, hepatic lipid metabolism largely determines triglycerides (TG) and cholesterol levels in the organism. In vivo studies showed an increased ERK1/2 activity in the liver of both, diet-induced and genetically obese mice [22]. Additionally, sustained hepatic ERK1/2 activity in the livers of lean mice perturbed glucose homeostasis, decreased energy expenditure and induced systemic insulin resistance [22]. Moreover, knocking down ERK1/2 expression in the liver of obese mice improved systemic insulin and glucose tolerance [22]. Consistent with these results, caloric restriction resulted in improved insulin sensitivity, which was accompanied by decreased hepatic activities of ERK1/2 kinases [23]. Indeed, obese *Erk1* knockout mice exhibited high degree of phosphorylation of liver’s MEK, the upstream regulator of ERK1/2. This phenomenon was associated with increased acetyl-CoA carboxylase 1 (ACC1) and fatty acid synthase (FAS) mRNA expression, indicating high lipogenesis, and decreased peroxisome proliferator-activated receptor α (PPARα) and carnitine palmitoyltransferase 1β (CPT1β) mRNA level suggesting a lower fatty acid (FA) oxidation [24].

Several potential pathways regulating systemic glucose and lipid metabolism, as well as insulin sensitivity via ERK1/2 were also further investigated. Constitutive activation of the ERK1/2 pathway was shown to suppress the expression of glucose-6-phosphatase (G6Pase) gene, a key enzyme in *de* novo glucose synthesis, and to decrease glucose output in hepatic cells [25]. The decreased expression of G6Pase gene is likely due to ERK1/2-mediated phosphorylation and cytosolic retention of the transcription factor Forkhead box O1 (FOXO1) [22]. Insulin-induced cytokine production in macrophages has also been implicated in the induction of insulin resistance in hepatocytes through ERK1/2 and Inhibitor of κB kinase β (IKKβ) activation. Mechanistically, activation of both kinases results in an inhibitory serine phosphorylation of the insulin receptor substrate (IRS) which blocks insulin action [26]. Additionally, fibroblast growth factor 21 (FGF21) was shown to act directly on the liver to stimulate phosphorylation of fibroblast growth factor receptor substrate 2 (FRS2) and ERK1/2. In vivo, acute FGF21 treatment induced hepatic expression of key regulators of gluconeogenesis, lipid metabolism, and ketogenesis [27]. Furthermore, overexpression of Hepassocin (HPS), a liver-derived protein, promotes lipid accumulation through an ERK1/2-mediated pathway [28], and recombinant HPS administration aggravates insulin signaling to induce insulin resistance through an ERK1/2-mediated signaling in hepatocytes [29]. In primary rodent hepatocytes, conjugated bile acids activate sphingosine-1-phosphate receptor 2 (S1PR2), which further activates the downstream ERK1/2 and AKT signaling pathways. Activation of ERK1/2 and AKT signaling pathway plays an important role in the regulation of hepatic glucose and lipid metabolism [30,31]. Moreover, p53 seems also to be involved in improving insulin sensitivity of hepatic cells through the inhibition of NF-κB and ERK1/2/p38 MAPK pathways [32]. However, the function of p53 with respect to insulin resistance appears to be highly controversial [32]. Similar to p53, serum- and glucocorticoid-regulated kinase 1 (SGK1) improves insulin sensitivity in the liver by inhibiting ERK1/2 activity [33]. 

Therefore, ERK1/2 kinases are the central regulators of hepatic glucose and lipid metabolism.

#### 2.1.2. ERK1/2 Promote Adipocyte Acquisition and Preserves Their Function

Adipose tissue is the central storage organ in the body. White, beige, and brown are the three major types of adipocytes, which play different physiological roles in whole-body energy homeostasis [34]. The main function of white adipocytes is to store excess energy as TGs for utilization during nutrient shortage. Beige and brown adipocytes dissipate the chemical energy stored in TGs as heat to preserve core temperature via the uncoupling of FA oxidation from ATP production, mediated by the uncoupling protein 1 (UCP1). During the time of increase, energy demand adipocytes can hydrolyze TGs into FAs and glycerol in the process of lipolysis. Lipolysis is tightly regulated by ERK1/2 [35]. In fact, ERK1/2 activity is significantly increased in adipose tissue of diet-induced obese mice. ERK1 deficient mice are protected from developing high-fat diet (HFD)-induced obesity, which is possibly due to impaired in vivo adipogenesis [36]. These observations are in contradiction to a recently published study showing that *Erk1**^−/−^*** mice, fed with HFD, were more obese than control animals [24]. HFD-induced obesity was associated with high fasting glucose and insulin concentrations, suggesting that a high degree of obesity in these animals might be caused, in part, by high insulin resistance. Though a plausible explanation for these observations is not available, it is worth noting the difference in FA composition of the diets used by both studies. In the genetic model of the obesity and hyperphagia (mice deficient for leptin function—*ob/ob* mice) deficiency of ERK1 results in partial protection from insulin resistance and liver steatosis due to the decreased adipose tissue inflammation and induction of muscle glucose uptake [37]. Interestingly, mouse primary adipocytes selectively express ERK2 but have undetectable levels of ERK1. Adipocyte-specific ERK2 knock-out mice exhibit decreased rates of lipolysis [35]. Mice with ERK2 inactivation or inhibition also fail to appropriately activate thermogenesis and maintain body temperature upon cold challenge due to lack of substrate availability. Indeed, ERK1/2 activation in adipocytes seems to be a critical regulatory step in the enhanced adipocyte lipolysis. Catecholamine-stimulated lipolysis in adipocytes is primarily a β-adrenergic and cAMP-dependent event. β3-adrenergic receptor (β3AR) regulates lipolysis through both, cAMP-dependent protein kinase A (PKA) and ERK1/2, by direct recruitment and activation of Src kinase [38]. A recent study showed that in vivo inhibition of the MEK/ERK pathway alters lipolysis in adipose tissue by decreasing β3-adrenergic receptor (β3AR) phosphorylation at serine 247 and subsequent downstream phosphorylation events that control the release of free FA (FFA) [35]. Furthermore, the activation of ERK1/2 by tumor necrosis factor α (TNFα) leads to increased basal lipolysis due to the downregulation of perilipin at mRNA and protein levels [39]. Perilipin coats lipid storage droplets in adipocytes, thereby protecting them until they can be broken down by hormone-sensitive lipase [40]. ERK1/2 also regulates lipolysis by the phosphorylation of hormone-sensitive lipase at Ser 600, increasing catalytic activity and the release of FFA from adipocytes [41]. Moreover, the activation of the Fas receptor, member of the TNF receptor, leads to ERK1/2-mediated lipolysis, which may be triggered by a Fas-induced increase in intracellular calcium levels and hence, autophosphorylation of the CaMKII [42].

ERK1/2 are also required in the early steps of adipogenesis [43] and need to be subsequently inhibited to prevent peroxisome proliferator-activated receptor γ (PPARγ) phosphorylation and to enhance terminal differentiation [44]. PPARγ is the main driver of adipocyte differentiation. PPARγ phosphorylation by ERK1/2 and JNKs results in decreased transcriptional activity, thereby attenuating differentiation [45].

FGF21 has anti-inflammatory effects on preadipocytes; these effects are mediated by the fibroblast growth factor receptor substrate 2/ERK1/2 signaling pathway [46]. Moreover, ERK1/2 seem also to be implicated in other mechanisms recently identified to induce adipogenesis, including the secretion of Caveolin-1 (Cav-1), a putative adipogenesis enhancer, from adipocytes [47], as well as the Fibrinogen-like-protein 1 (FGL1)/ CCAAT/enhancer-binding protein β (C/EBPβ) pathway [48]. Finally, Erk1/2 present also critical roles in promoting norepinephrine (NE)- and basic fibroblast growth factor (bFGF)-dependent survival of brown adipocytes [49] and in browning of white adipocyte [50]. Angiotensin type 2 receptor (AT2R) stimulates white adipocyte browning through ERK1/2, leading to enhanced UCP1 expression and exhibition of brown-like phenotypes [50].

To sum up, ERK1/2 promote adipogenesis, lipolysis, and thermogenesis in different depots of adipose tissue.

#### 2.1.3. ERK1/2 Promote Inflammation during Obesity

Obesity induces infiltration of immune cells, promoting chronic low-grade inflammation and eventually leading to adipocyte death. Moreover, circulating proinflammatory cytokines, and FFAs derived from the inflamed adipose tissue, impair insulin sensitivity in the periphery, which is the major cause for the development of T2D [51]. Macrophage-elicited metabolic inflammation and adipocyte-macrophage interaction are in fact key factors in obesity [52]. Conventional MAPKs, including ERK1/2, JNKs, and p38s, are essential regulators of adipose tissue inflammation, linking TNFα signaling to activation of downstream transcriptional programs that promote pro-inflammatory gene expression [53,54]. While the critical role of both ERK1 and ERK2 in the process of positive selection and maturation of CD4+ and CD8+ T cell [55] and macrophage development [56] is well established, there is also supporting evidence that underlays their role in metabolic inflammation. In the context of macrophage-adipose cell interactions, several studies revealed that macrophage-conditioned medium (MaCM), collected under basal conditions, protects preadipocytes from apoptosis in a platelet-derived growth factor (PDGF)-dependent manner [57,58]. MaCM-induced preadipocyte survival depends on signaling through Akt, ERK1/2, and reactive oxygen species (ROS) [58]. ERK1/2 act also as an important signaling mediator for the inhibitory effect of MaCM on TG accumulation during adipogenesis [59]. Of note, FGF21 acts on adipocytes in an autocrine manner to promote the expression and secretion of chemoattractant C-C motif chemokine ligand 11 (CCL11) via activation of ERK1/2, which drives recruitment of eosinophils into subcutaneous white adipose tissue (WAT), leading to increased accumulation of M2 macrophages, and proliferation and commitment of adipocyte precursors into beige adipocytes [60]. Dual specificity phosphatases (DUSPs), which are ERK1/2 phosphatases, inactivate ERK1/2 through dephosphorylation and can thus inhibit inflammatory gene expression. For example, in epididymal WAT and in response to diet-induced obesity, DUSP5 functions as the feedback regulator of TNFα-evoked ERK1/2 signaling, which results in increased inflammatory gene expression [61]. Finally, inflammation induced by macrophage-derived cytokines, interleukin-1β (IL-1β) and TNFα, could suppress the induction of UCP1 expression in adipocytes through ERK1/2 activation [62,63]. 

Taken together, ERK1/2 determine inflammatory response during obesity.

#### 2.1.4. ERK1/2 Drive Insulin Production in Pancreatic β-Cells

Diabetes is a complex metabolic disorder caused by insufficient insulin action in the peripheral tissues and defective insulin secretion from the pancreatic β-cells. Insulin is the most potent hormone promoting TG synthesis and inhibiting lipolysis [64]. Several studies have suggested a positive regulatory role of the ERK1/2 signaling in glucose-stimulated insulin secretion and β-cell survival [65,66]. Physiological concentrations of glucose that lead to Ca^2+^ entry and insulin secretion activate ERK1/2 in pancreatic β-cell [67], where they are required for insulin gene transcription [68] and insulin release from pancreatic β-cells and rodent pancreatic islets [69]. Interestingly, however, it is ERK1 specifically, and not ERK2 that seems to be necessary for glucose-induced full activation of key proteins involved in β-cell function, namely MSK1 and CREB [70]. Indeed, MSK1 and CREB activities were restored when ERK1 was reintroduced in *Erk1^−/−^* mouse β-cells, but not when ERK2 was overexpressed, which indicates a specific role for ERK1 in these processes. However, ERK2 activity is likely to be sufficient for insulin gene expression and the first phase of glucose-stimulated insulin secretion [70]. The ERK1/2 signaling module is typically activated by an upstream small G-protein activation (Ras), which in turn activates Raf (MAPKKK) and MEK1/2 (MAPKK). Glucose-induced ERK1/2 activation was inhibited in pancreatic β-cells expressing kinase-negative mutants of Ras and Raf kinases [71]. However, Raf-MEK-ERK signaling axis can be also activated by Rap1, another small GTPase, in human pancreatic β-cells upon stimulation with glucose and GLP-1 [72]. Moreover, increased concentrations of FAs in blood, and in particular saturated FAs (e.g., stearic and palmitic acid), are known to be one of the main factors responsible for pancreatic β-cell death in T2D [65]. The effects of saturated FAs on ERK1/2 activity and their pro-apoptotic or pro-survival role in β-cells tend to be rather contradictory [65]. However, in the presence of MEK1 inhibitor, the activity of adiponectin, a pancreatic β-cells pro-survival hormone secreted by adipocytes, was blocked. These findings suggest pro-survival function of ERK1/2 module in pancreatic β-cells [73].

Taken together, ERK1/2 promote pancreatic β-cell mass and insulin secretion.

#### 2.1.5. ERK1/2 Promote Skeletal Muscle Acquisition and Metabolism

Skeletal muscle is the primary site for glucose uptake and storage in the form of glycogen, and, accordingly, plays a key role in the control of the whole body glucose metabolism [74]. Insulin and muscle contraction activate conventional MAPKs (ERK1/2, JNKs, and p38s) signaling in skeletal muscle [75]. Insulin-mediated responses on MAPK signaling are impaired in skeletal muscle from obese mice, whereas the effect of contraction is generally well preserved [76]. Animals lacking muscle ERK1/2 displayed impaired postnatal growth, muscle weakness, and a shorter life span [77]. Their muscles displayed fragmented neuromuscular synapses and a mixture of modest fiber atrophy and loss. On the other hand, muscle atrophy during cachexia was associated with mitochondrial depletion and up-regulation of ERK1/2 and p38 MAPKs activity [78]. Moreover, blocking of the activity of ERK1/2 pathways by using MEK1 inhibitors prevents chemotherapy-related cachexia [78]. Altogether, these data indicate that ERK1/2 promote developmental processes in muscles, but these kinases also mediate pathological processes in muscular fibers evoked medications used during chemotherapy.

ERK1/2 are involved in various metabolic mechanisms at skeletal muscle level. Palmitic acid (PA)-mediated glucose uptake in skeletal muscle cells is stimulated by the binding of PA to cell surface and followed by activation of PI3K/ERK1/2 pathway [79]. Furthermore, complement C1q/TNF-related protein 6 (CTRP6) mediates lipogenesis in myoblasts through AdipoR1/ERK1/2/PPARγ signaling pathway [80]. ERK1/2 pathway negatively regulates glycogen synthase (GS) activity in myotubes, independently of Glycogen synthase kinase 3 (GSK3) [81]. ERK1/2 are also required to phosphorylate PH domain and leucine rich repeat protein phosphatase 1α (PHLPP1α) at Ser932 under endoplasmic reticulum (ER) stress, which is required for its ability to interact with and dephosphorylate AMPK and thereby induce ER stress [82]. PHLPP1 expression is enhanced in the skeletal muscle of insulin resistant rodents that also exhibit ER stress, an important mediator of insulin resistance. 

Therefore, ERK1 and ERK2 promote muscle differentiation and sustain their metabolism.

#### 2.1.6. The Central Role of ERK1/2 in Regulation of Appetite and Energy Dissipation

The central nervous system (CNS) integrates signals generated by the adipose tissue-, pancreas-, and gastrointestinal-derived hormones to regulate energy homeostasis. In the CNS, insulin and leptin are the key hormones that act on hypothalamic neurons to decrease food intake and hepatic glucose production and to increase energy expenditure [83]. The hypothalamus, and particularly the arcuate nucleus (ARC), is central to the convergence of nutrient signals (e.g., glucose). ARC consists of two neuronal populations that modulate food intake and energy expenditure [84]. Neurons leading to a positive energy balance express agouti-related protein (AgRP) and neuropeptide Y (NPY), while neurons leading to a negative energy balance express proopiomelanocortin (POMC). In response to glucose, ERK1/2 regulate POMC but not AgRP/NPY expression in hypothalamic neurons [85]. Fasting activates ERK1/2 in the ARC and the paraventricular nucleus in mice [86,87] and the activation is reversed by refeeding [87]. Obesity is associated with increased protein levels of hypothalamic MAPK phosphatase-3 (MKP-3), which is related to the reduction of ERK1/2 phosphorylation in the hypothalamus as well as to an increase in body weight and a reduction in energy expenditure [88]. In parallel, leptin acts on the hypothalamus through its receptor, increasing the phosphorylation (Thr202/Tyr204) of ERK1/2 [89,90]. Mechanistically, leptin binds to the extracellular domain of the leptin receptor (LepR) dimer and activates the Jak2 tyrosine kinase. Activated Jak2 tyrosine phosphorylates itself and Tyr985 and Tyr1138 on the intracellular tail of LepR. Phosphorylated Tyr985 recruits the SH2-containing tyrosine phosphatase (SHP-2), which is itself phosphorylated and binds growth factor receptor binding 2 (Grb-2) to activate ERK1/2 signaling pathway. Inhibition of ERK1/2 reduces the ability of leptin to stimulate the sympathetic signal of the hypothalamus to brown adipose tissue, resulting in lower heat production [89]. Acute physical exercise also increases leptin-induced hypothalamic phospho-ERK1/2, associated with higher BAT UCP1 content and heat production in obese mice [91]. The increase in ERK1/2 phosphorylation is likely to depend on the intensity of the physical exercise. Therefore, physical activity promotes leptin-dependent activation of Erk1/2 in the hypothalamus to drive thermogenesis. Moreover, hepatocyte-secreted hormone FGF21 induces fasting gluconeogenesis via the brain-liver axis. FGF21 acts directly on the hypothalamic neurons to activate ERK1/2, thereby stimulating the expression of corticotropin-releasing hormone by activation of the transcription factor CREB [92]. In addition to the hypothalamus, the dorsal vagal complex (DVC) is an extra-hypothalamic region that integrates nutritional and hormonal signals to regulate peripheral metabolism. Insulin triggers a PI3K-independent and ERK1/2-dependent signaling cascade in the DVC to lower glucose production in healthy rodents [93]. Indeed, acute insulin infusion into the DVC activates ERK1/2 to lower food intake in healthy but not high-fat diet fed rats [94]. Direct molecular disruption of DVC ERK1/2 signaling in normal rats induces hyperphagia and obesity, whereas daily acute repeated DVC insulin infusion lowers food intake and body weight in normal rats [94]. Besides that, ERK1 also regulates feeding behaviors. *Erk1* knock-out mice have been shown to exhibit low preference for dietary fat [95]. Mechanistically, in the taste bud cells ERK1/2 is activated by the opening of a specific calcium channel, calcium-homeostasis modulator-1 (CALHM1), to modulate orogustatory detection of dietary lipids in mice and humans.

All of these indicates that induction of ERK1/2 signaling in hypothalamus suppresses appetite and promotes energy expenditure in the periphery.

#### 2.1.7. Summary

**Figure 1:**
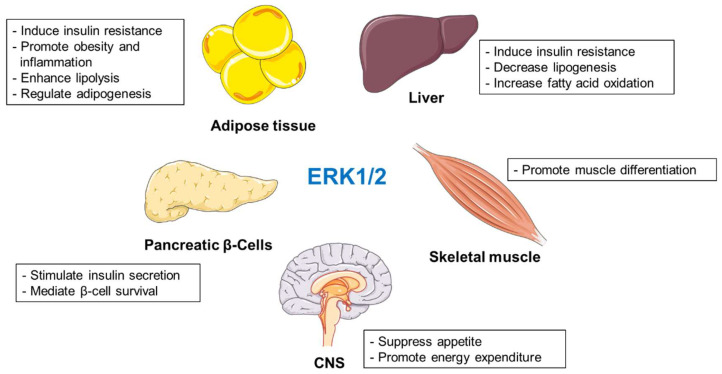
Roles of ERK1/2 in regulation of metabolism.

Different studies have shown that ERK1/2 regulate several metabolic events, and their activation is associated with deleterious effects during obesity and diabetes (Figure 1). However, several reports indicate that inactivation of ERK1/2 might promote or suppress the development of obesity and insulin resistance depending on the animal models used in the given studies [24,36,37]. Nevertheless, deletion of ERK1/2 in the liver improved systemic insulin and glucose tolerance. [22]. Interestingly, mouse primary adipocytes selectively express ERK2 [35]. Adipocyte-specific ablation of ERK2 resulted in decreased rates of lipolysis and fail to appropriately activate thermogenesis. ERK1/2 also promote adipogenesis [43,44] and determine inflammatory response during obesity [58,59,60,61,62,63]. Moreover, ERK1/2 positively regulate glucose-stimulated insulin secretion and β-cell survival [65,66]. Animals lacking muscle ERK1/2 displayed muscle weakness with a mixture of modest fiber atrophy and loss [77]. In hypothalamus, ERK1/2 suppress appetite and promote energy expenditure in the periphery [89,91,94].

### 2.2. JNK Kinases

The cJun NH2-terminal kinase (JNK), also known as stress-activated protein kinase (SAPK), has three isoforms JNK1, JNK2, and JNK3 that are encoded by three separate genes. Whereas JNK1 and JNK2 are ubiquitously expressed in mammalian cells, JNK3 is predominantly expressed in brain, testis, and heart [96]. The JNK proteins are activated by a variety of extracellular stimuli, including stress (hypoxia, UV, and ionizing radiation), cytokines, growth factors, pathogens, toxins, drugs, and metabolic changes, including obesity and hyperlipidaemia. JNKs are directly activated by two upstream MAPKK enzymes, MKK4 and MKK7. Activation of JNK isoforms requires dual phosphorylation on Thr and Tyr residues within a conserved Thr-Pro-Tyr motif in their activation loops. Several enzymes, such as MEKK1 to –4, MLK1/2/3, Tpl-2, DLK, TAO1/2, TAK1, and ASK1/2, have been reported to act as MAPKKKs and consequently activate JNKs. Many substrates have been shown to be phosphorylated by JNKs (reviewed in [2,96,97]).

Through the phosphorylation of their targets (Table 1), JNKs play an important role in the control of apoptosis, cell proliferation, and cell migration [2]. JNKs phosphorylate and activate c-jun, a member of the AP-1 transcription factor family [98]. JNK1/2 promote the formation of a complex between c-jun and other members of the family of AP-1 transcription factors and thereby, mediate the transcription of AP-1 target genes involved in cell cycle and apoptosis. JNKs are known to also induce apoptosis by interacting with its substrate proteins of the outer mitochondrial membrane, including BH3-only family of Bcl2 proteins. [99,100]. Additionally, JNK1/2 phosphorylate other substrate proteins including p53, ATF-2, NF-ATc1, Elk-1, HSF-1, STAT3, c-Myc, and JunB [2,66]. 

The JNK pathway is also implicated in the regulation of obesity, T2D, insulin resistance, and atherosclerosis [4,5,6,101,102]. JNK activity is significantly elevated in various tissues, such as liver, muscle, and fat, in T2D patients and in diet-induced and genetic animal models of obesity and diabetes [103,104,105,106]. Mice lacking JNK1, but not JNK2, are protected against obesity and insulin resistance [103]. On the other hand, the deletion of JNK2, but not JNK1, protects from the development of atherosclerosis on in ApoE**^−/−^** mice [102]. The JNK2 isoform is also involved in metabolic regulation, but its function is not evident when JNK1 is fully expressed due to the regulatory crosstalk between the two isoforms [107].

#### 2.2.1. Functions of JNKs in the Liver

In hepatic-specific JNK-deficient mice, or in animals in which deficiency of JNK1 was induced by adenoviral delivery of shRNA [108], or mice expressing a dominant-negative JNK [109] insulin resistance evoked by HFD feeding was ameliorated. This was associated with up-regulation of the hepatic expression of clusters of genes regulating glycolysis and several genes involved in the triglyceride synthesis pathways [109]. Moreover, hepatocyte-specific double knockout of JNK1 and JNK2 induced systemic protection against HFD-induced insulin resistance [110]. These effects were associated with an upregulation of nuclear hormone receptor PPARα and disruption of the hepatic PPARα/FGF21 hormone axis, leading to marked increases in the rate of FA oxidation, ketogenesis, and improved hepatic insulin action. JNKs modulate liver insulin resistance mainly through insulin receptor adapter protein IRS1 phosphorylation on serine 307. Upon activation by proinflammatory cytokines (e.g., TNFα) and circulating FFAs, JNKs prevent its interaction with the insulin receptor and cause insulin resistance [111]. However, mice in which Ser 307 was replaced by alanine showed reduced insulin sensitivity [112]. Interestingly, insulin stimulation itself leads to Ser 307 phosphorylation of IRS1, particularly in hepatocytes [112]. These data were supported by another report demonstrating that mice with hepatocyte-specific deletion of JNK1 displayed increased insulin resistance, glucose intolerance, and hepatic steatosis, showing that JNK1 plays a protective role in hepatocytes [113]. More recently, JNK-mediated regulation of adipokines [114] and inflammatory cytokines [115] has been implicated in the development of insulin resistance. Since the promotion of hepatic insulin sensitivity caused by JNK-deficiency in adipocytes and myeloid cells is associated with defects in adipokine/cytokine expression, it is possible that obesity-induced activation of JNK in non-hepatic cells mediates HFD-induced hepatic insulin resistance [114,115]. 

Overall, these findings from separate laboratories indicate that hepatic JNKs play a crucial role in the regulation of insulin sensitivity as well as glucose and lipid metabolism.

#### 2.2.2. JNKs Promote Inflammatory Mediators in Adipose Tissue

Deletion of JNK1 specifically in adipocytes improved adipose tissue insulin action and suppressed liver insulin resistance induced by high-fat diet feeding [114]. Improvement in hepatic insulin sensitivity evoked by deletion of JNK1 in adipocytes was associated with a reduction in the expression of the inflammatory cytokine IL-6 secreted by adipose tissue, and the expression of the classical IL-6 target gene, suppressor of cytokine signaling 3 (SOCS3), in the liver. SOCS3 has previously been linked to the development of hepatic insulin resistance [116]. Similarly to ERK1/2, the activation of TNFα leads to increased basal lipolysis due to the downregulation of perilipin mRNA and protein [39]. Moreover, high mobility group box 1 (HMGB1), a pro-inflammatory adipocytokine involved in WAT inflammation and insulin resistance in patients with obesity was secreted by adipocytes in response to JNK signaling [117]. Furthermore, the scaffold protein JNK interacting protein 1 (JIP1) has been shown to play a central and cell-specific role in JNK activation in the adipose tissue. Mice lacking JIP1 are protected from diet-induced obesity and show less severe insulin resistance. JNK cannot be activated in the adipose tissue and muscle of these mice, preventing them from the development of insulin resistance in both organs. However, livers of these animals have no defect in JNK activation [118]. Finally, blocking JNK activation prevented cold-induced subcutaneous WAT beiging [119]. ERK 1/2 and JNK were both activated in beige adipocytes under ER stress conditions and regulated the mRNA levels of UCP1 involved in thermoregulation and PPARγ, which is known as a UCP1 transcriptional activator [120]. Interestingly, only JNK inhibition, but not ERK, rescued the PPARγ protein. 

In summary, JNKs promote insulin resistance, inflammation, and beige adipocytes in adipose tissue.

#### 2.2.3. The role of JNKs in Immune Cells

Early studies indicate that JNKs may also be involved in obesity-induced inflammation [107]. Reciprocal adoptive transfer experiments demonstrated that JNK1 deletion in non-hematopoietic tissues protects against weight gain and, partly as a consequence, from insulin resistance [121]. On the other hand, hematopoietic JNK1 removal reduces obesity-induced inflammation and protects against HFD-induced insulin resistance without affecting obesity [121]. However, another study indicated that JNK1-deficient bone marrow transplantation was insufficient to affect macrophage infiltration or insulin sensitivity despite minimal changes in the inflammatory profile of adipose tissue, whereas this protection was induced by the absence of JNK1 in parenchymal cells [122]. Importantly, HFD-fed mice overexpressing dominant-negative JNK in adipose tissue and macrophages showed reduced weight gain, insulin resistance, glucose intolerance, and hepatic steatosis. These mice had smaller than normal adipocytes, reduced macrophage infiltration in adipose tissue, and less severe whole-body inflammation, highlighting an important role for JNK in the crosstalk between adipose tissue and macrophage infiltration [123]. Indeed, the combined loss of both JNK1 and JNK2 from macrophages results in a significant protection from obesity-induced impairments to glucose metabolism and the development of insulin resistance but has no effect on HFD-induced obesity [115]. The myeloid JNK1/2-knockout mice also show less macrophage infiltration and decreased M1 polarization in adipose tissue. M1 polarization was also decreased in hepatic macrophages, accompanied by increased M2 polarization, highlighting an important role for JNKs in M1 polarization. Macrophages polarization to the M1, pro-inflammatory state leads to enhanced production of pro-inflammatory factors such as IL-1β, TNFα, and IL-6. Thus, in the context of a HFD, pro-inflammatory cytokines within the adipose tissue are likely to contribute to elevated lipolysis by directly initiating it, and by impairing insulin ability to suppress it [124]. The consequence of this inflammation-dependent increase in adipose tissue lipolysis is elevated hepatic acetyl-CoA content, which allosterically activates pyruvate carboxylase, leading to the generation of oxaloacetate and subsequent conversion to glucose. Remarkably, in mice fed a HFD, the deletion of JNK from macrophages reduces hepatic acetyl-CoA content, pyruvate carboxylase activity and dramatically improves the ability of insulin to suppress hepatic glucose production [124]. These data clearly associate inflammation in adipocyte tissue to both fasting and postprandial hyperglycemia in T2D. Finally, non-obese diabetic mice lacking JNK2 exhibit a decrease in destructive insulitis and a reduced disease progression to diabetes, probably due to increased Th2 polarization of CD4+ T cells [125]. This study reported that T-cell polarization effect was not seen in JNK1**^−/−^** mice, indicating a specific role for JNK2 in the regulation of T-cell polarization in type 1 diabetes [125]. 

Taken together, JNK1/2-mediated signaling in immune cells promotes inflammation, which then mediates peripheral insulin resistance as well as pancreatic β-cell death.

#### 2.2.4. The Impact of JNKs on Pancreatic β-Cells during Type 1 and Type 2 Diabetes

Different studies have demonstrated that inhibition of JNKs prevents β-cell dysfunction and apoptosis in human islets and in β-cell lines [126,127,128,129]. Indeed, in a selective in vivo model of β-cell dysfunction, inhibition of JNK using either JNK inhibitor or JNK1-null mice preserves β-cell function during hyperglycemic clamps [130]. Mechanistically, chronically elevated concentrations of leptin and glucose induce β-cell apoptosis through the activation of the JNK pathway [128]. Type I and II interferons (IFNs) production may interact with protein tyrosine phosphatase non-receptor 2 (PTPN2) to induce aberrant proapoptotic activity of the BH3-only protein Bim, leading to an increased β-cell apoptosis via JNK activation and the intrinsic apoptotic pathway. JNK1/2 mediates IL-1β-induced ER Ca^2+^ release and the consequent mitochondrial dysfunction in mouse and primary human pancreatic β-cells [131]. Oxidative stress-induced activation of JNK impairs insulin signaling cascade [132] and thus activates FOXO-1, resulting in decreased expression and nuclear localization of pancreatic and duodenal homeobox 1 (Pdx-1) with consequent reduction of insulin gene transcription. Finally, JIP1 has been also shown to mediate β-cell apoptosis [133]. However, studies in animals lacking JIP1/JIP2 proteins indicate that these proteins are not essential for β-cell viability [134]. Importantly, several studies have proposed that JNK isoforms exhibit differential roles in β-cell apoptosis depending on the stimuli. JNK3 was found to have antiapoptotic properties in cytokine-induced β-cell apoptosis, whereas JNK1 and JNK2 were reported to serve proapoptotic functions in response to cytokines [135]. JNK1, however, exerts an antiapoptotic function in palmitate- and high glucose-induced β-cell death [136]. JNK2 knockdown did not influence palmitate- and high glucose-induced β-cell apoptosis, and JNK3 knockdown only increased cleaved caspase 9 and 3 but not apoptosis [136]. In addition, FFA stimulates autophagy in β-cells via JNK1 signaling pathways independently of oxidative or ER stress, and JNK2**^−/−^** β-cells in non-obese mice are protected against apoptosis induced by T cells in a model of autoimmune type 1 diabetes [125]. 

Therefore, specific members of JNK family differentially regulate pancreatic β-cell apoptosis depending on the stimuli.

#### 2.2.5. JNKs in Skeletal Muscle Metabolism

Similar to other tissues, JNK1 is activated in muscle from HFD-fed mice and, therefore, may potentially impact skeletal muscle glucose metabolism [103]. Early studies showed that overexpression of a constitutively active (CA) JNK, but not WT JNK, in the tibialis anterior muscle of mice led to a reduction in insulin-stimulated glucose clearance [137]. However, no effect of skeletal muscle-specific CA JNK on the development of obesity, glucose tolerance, or insulin sensitivity could be observed [138]. Moreover, inhibiting JNK in muscle by overexpressing heat shock protein 72 (HSP72) improved HFD-induced hyperglycaemia and hyperlipidaemia [104]. Furthermore, it prevented glucose intolerance and insulin resistance and reduced HFD-induced weight gain. However, skeletal muscle-specific deletion of JNK1 did not affect the development of HFD-induced obesity [138,139]. Yet, these mice were protected against obesity-induced insulin resistance and showed increased glucose uptake by muscle. Additionally, adipose tissue and liver were not positively affected by JNK1 deficiency in skeletal muscle. Instead, these mice showed increased hepatic steatosis and circulating levels of triglycerides and increased adipose tissue macrophage infiltration [139]. In contrast, no effect of skeletal muscle-specific JNK1 deletion on any indices of glucose metabolism or insulin sensitivity was observed in a similar study using the same in vivo model [138]. Finally, ER stress induced by palmitate could increase the expression of hepassocin (HPS) in hepatocytes and further contribute to the development of insulin resistance in skeletal muscle via EGFR/JNK-mediated pathway [140]. 

Therefore, JNK-mediated signaling in muscle likely attenuates glucose uptake in this tissue but has a minor effect on the whole-body insulin sensitivity.

#### 2.2.6. Central Regulation of Metabolism by JNKs

JNKs activity was significantly elevated in the hypothalamus [83,141] and pituitary gland [83] in obese mice. In CNS/pituitary-specific JNK1 knockout mice, a reduced body-weight gain was observed and correlated with an increased energy expenditure and locomotor activity and a higher body temperature [83,142]. These mice were protected against insulin resistance and glucose intolerance at the central and peripheral levels. Moreover, they displayed activation of the hypothalamus-pituitary-thyroid axis, as indicated by increased circulating thyroid-stimulating hormone (TSH), T3, and T4 levels, as well as increased expression of TSHβ and the thyroid releasing hormone receptor (TRHR) within the pituitary gland. Collectively, these data indicate a major role of JNK1 in the control body homeostasis by regulating the pituitary-thyroid axis [83,142]. These observations were supported by another report showing that pituitary-specific deletion of JNK1 and JNK2 largely prevented HFD-induced obesity [143]. These mice have elevated circulating levels of T3, T4, and TSH. In fact, JNK1/2 regulate expression of type 2 iodothyronine deiodinase (Dio2), which encodes the enzyme responsible for T4 to T3 conversion in the pituitary gland. Impairment of this conversion reduces the negative feedback regulation of the expression of TSH, resulting in sustained production of TSH, T3, and T4 [143]. In contrast, mice expressing constitutively active JNK1 in AgRP-expressing neurons become more obese and are more leptin resistant at the neuronal and systemic levels than control animals when fed a HFD [141]. Indeed, AgRP-specific deletion of p53 resulted in increased hypothalamic JNK activity before the mice developed obesity, and central inhibition of JNK reversed the obese phenotype of these mice [144]. Mechanistically, T3 regulates hepatic lipogenic pathway by increasing AMPK-induced JNK1 activity specifically in the ventromedial nucleus of the hypothalamus (VMH), leading to hepatic vagal activation [145]. 

Overall, hypothalamic activation of JNK pathway promotes obesity and diabetes.

#### 2.2.7. Summary

**Figure 2:**
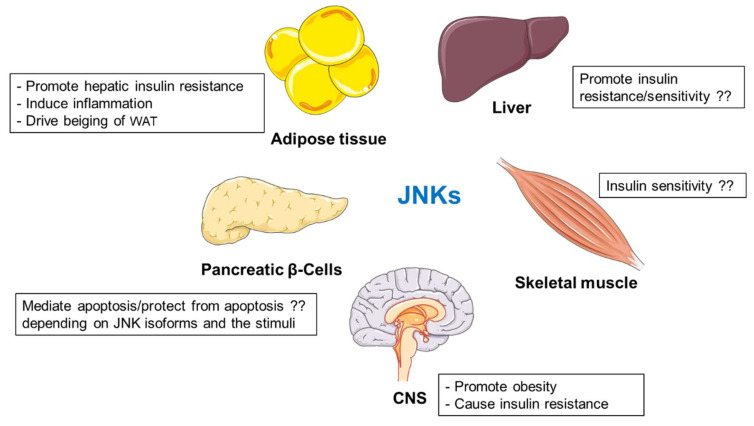
The role of JNKs in various tissues and organs.

JNKs are central kinases regulating metabolism in multiple organs (Figure 2). Mice lacking JNK1, but not JNK2, are protected against obesity and insulin resistance [103]. JNK2 is also involved in metabolic regulation, but its function is not evident when JNK1 is fully expressed. [107]. Findings from separated laboratories present an ambiguous picture of the role of hepatic JNKs in regulation of metabolism [108,109,110,111,112,113]. However, JNK-deficiency in non-hepatic cells (adipocytes and myeloid cells) promotes insulin sensitivity [114,115]. Deletion of JNK1 specifically in adipocytes improved adipose tissue insulin action but prevented cold-induced subcutaneous WAT beiging [114,119]. Moreover, JNK1/2-mediated signaling promotes inflammation [107,115,121,122,123,124,125]. Additionally, JNK isoforms present differential roles in pancreatic β-cell, promoting or suppressing apoptosis depending on the stimuli [135,136]. Different contradictory results do not fully elucidate the role of JNK proteins expressed in skeletal muscle [104,137,138,139]. Finally, JNK activation in hypothalamus contributes to the development of obesity and diabetes [83,142].

### 2.3. p38 Kinases

There are four members of the p38 MAPKs family (p38α, β, γ, and δ) encoded by four different genes that have different tissue expression patterns. p38 isoforms are expressed differently in individual cells and have varied and often opposite effects even within the same cell on the same substrate [2,6]. p38 isoforms are strongly activated by various environmental stresses including oxidative stress, UV irradiation, hypoxia, ischemia, inflammatory cytokines, GPCRs, and Rho family GTPases. In addition to the usual activation by two upstream MAPKKs, MKK3 and MKK6, the p38 MAPKs can also be activated by binding of TAB1 or phosphorylation by the tyrosine kinases ZAP70 and LCK. MKK3/6 are activated by several MAPKKKs, including MEKK1 to –3, MLK2/3, ASK1, Tpl-2, TAK1, and TAO1/2 [2]. All p38 isoforms are activated in response to appropriate stimuli by dual phosphorylation in the activation loop sequence Thr-Gly-Tyr. 

p38 MAPKs play critical roles in a wide variety of cellular processes such as proliferation, differentiation, regeneration, and metabolism [2]. Upon stimulation, p38 isoforms phosphorylate a large number of substrates in many cellular compartments (Table 1) (reviewed in [2,146]). Similarly, to ERK1/2, p38s are known to interact and phosphorylate MSKs and MNK1/2 [10,11]. These kinases are involved in multiple biological functions including cell differentiation, cytokine production, cell cycle regulation, and apoptosis [10,11]. Furthermore, p38s are also known to phosphorylate transcription factors including ATF, Elk-1, and p53 [66]. P38 MAPKs phosphorylate and stabilize p53 by preventing its MDM2-induced proteasomal degradation, resulting in its nuclear accumulation and the transcription activation of its apoptotic target genes [147,148]. On the other hand, p38s-ATF axis was shown to regulate ER stress response [149,150]. Additionally, p38s are involved in inflammatory responses by interacting with nuclear transcription factors such as ATF-2 and NF-κB, resulting in the expression of inflammatory cytokines [151].

p38α, which is by far the best-characterized member of the p38 family, is known to phosphorylate several key proteins involved in glucose and lipid metabolism [6]. As most commonly used p38 inhibitors (SB203580 and SB202190) targets only p38α and β isoforms [152], relatively less is known about p38γ and δ isoforms. However, many studies suggest that p38s regulate the metabolism of different cell types and are central in the pathogenesis of metabolic diseases.

#### 2.3.1. The Role of p38s in the Liver

Hepatic p38 is a pivotal regulator of hepatic gluconeogenesis. It has been shown that liver expression of dominant-negative p38α in obese mice reduced fasting insulin levels and improved glucose tolerance [153]. In response to different stress stimuli, p38α activation initiates ErbB receptors (epidermal growth factor EGF/ErbB family of receptor Tyr-kinases) signaling, leading to Ser phosphorylation of IRS1 proteins which impair cellular response to insulin [153]. Indeed, mice lacking hepatic p38α exhibited reduced fasting glucose levels and impaired gluconeogenesis [154]. In addition, an elevated liver expression of p38δ was detected in a cohort of obese patients with NAFLD, and p38γ and p38δ were found to be responsible for the development of steatosis in different mouse models of NAFLD [155]. p38α stimulates hepatic gluconeogenesis by regulating the phosphorylation of CREB and the expression of PGC-1α [156]. PGC-1α has been identified as a direct target of p38α/β [157]. PGC-1α also co-activates many other transcription factors, including the glucocorticoid receptor, FOXO1, and PPARα. Phosphorylation of the glucocorticoid receptor by p38α/β seems to enhance its transcriptional activity [158], whereas phosphorylation by JNKs and ERK1/2 inhibits its function [159,160]. The transcriptional activity of PPARα is also enhanced by phosphorylation by ERK1/2 and p38α/β [161]. Moreover, CREB is a direct target of MSK that is activated by ERK1/2 and p38α/β [162]. However, hepatic deficiency of p38α in vivo did not result in an alteration in CREB phosphorylation, suggesting that p38α signaling might not be required for the activation of CREB during fasting [154]. Furthermore, p38α/β phosphorylate CCAAT-enhancer-binding protein-α (C/EBPα) [163], a transcription factor that regulates hepatic gluconeogenesis [164]. Phosphorylation on serine 21 enhances C/EBPα transactivation activity and increases phosphoenolpyruvate carboxykinase kinase (PEPCK), a gluconeogenic gene, expression [163]. p38α/β also phosphorylate the spliced form of X-box binding protein 1 (Xbp1s), promoting its nuclear translocation, reducing ER stress, and improving glycemia during obesity [165]. Moreover, p38α/β were shown to be required for glucagon and fasting-mediated suppression of hepatic lipogenesis, possibly through the inhibition of central lipogenic genes transcription, sterol regulatory element-binding protein 1 (SREBP1) and PGC-1β [166]. A more recent study showed that DUSP12, which plays an important role in brown adipocyte differentiation, physically binds to ASK1, promotes its dephosphorylation, and inhibits its action on p38α/β in order to reducelipogenesis and to suppress lipid accumulation in livers of high-fat fed mice [167]. Similar to DUSP12, DUSP14, DUSP26 and MKP-5 suppress the development of hepatic steatosis by inhibiting p38s [167,168,169,170]. Both DUSP14 and DUSP26 directly bind and dephosphorylate TAK1 kinase, which results in the inhibition of TAK1 and its downstream targets JNKs and p38s [168,169]. MKP-5 prevents the development of hepatic steatosis by suppressing p38–ATF2 and p38–PPARγ signaling axis to reduce hepatic lipid accumulation [170]. Finally, F-prostanoid receptor (FP) activation through the CaMKIIγ/p38/FOXO1 signaling pathway was demonstrated to facilitate hepatic gluconeogenesis in mice by upregulating gluconeogenic genes under both fasting and diabetic conditions [171]. FP activation promoted FOXO1 nuclear translocation by stimulating CaMKIIγ signaling, and p38 was required for this translocation. 

Taken together, members of p38 family promote hepatic insulin resistance and gluconeogenesis but suppress lipogenesis in liver.

#### 2.3.2. Functions of p38s in Adipose Tissue

p38s have long been known as a central mediator of cAMP/PKA signaling, which positively regulate the transcription of UCP1 in brown adipocytes [172]. Besides, the p38α/β downstream target activating transcription factor 2 (ATF2) induces the expression of PGC-1α, and these two nuclear transcription factors together control the expression of UCP1 [172]. In WAT, the activation of p38α could also be mediated by the alternative mechanism involving AMPK, TAK, and TAB [173]. Similarly, bone morphogenetic protein 7 (BMP7) is able to activate a full program of brown adipogenesis, including UCP1, via p38α/β-dependent pathways [174]. Additionally, irisin, a hormone secreted by skeletal muscles, promotes WAT browning by stimulating expression of WAT browning-specific genes via the p38α/β and ERK1/2 pathways [175]. Surprisingly, adipocyte-specific deletion of p38α caused minimal effects on BAT in adult mice, as evident from undetectable changes in UCP1 expression, mitochondrial function, body temperature, and energy expenditure [176]. On the other hand, genetic ablation of p38α in adipose tissues not only significantly facilitates the browning in WAT upon cold stress, but also prevents diet-induced obesity [176]. Mechanistically, inhibition of p38α stimulates the UCP1 transcription through PKA and its downstream target CREB [176]. However, these observations are in contradiction with a recently published study using the same in vivo model, and showing that adipocyte-specific p38α knockout mice were protected against HFD-induced obesity, had increased energy expenditure and a higher BAT thermogenesis [177]. Lack of p38α in BAT resulted in higher activation of p38δ, suggesting that p38α controls p38δ activity, thereby regulating thermogenesis and energy homeostasis. In contrast, in WAT, p38α would have opposite effects depending on the fat depots, blocking browning through inhibition of p38γ in inguinal fat (iWAT) [177], and promoting browning in epididymal fat (eWAT) [173]. Moreover, β3-adrenergic-induced p38 and JNK pathways promote hormone-sensitive lipase (HSL)-mediated lipolysis. At the same time, p38α/β and JNKs mediate pro-inflammatory cytokines expression in adipose tissue [178]. Finally, it has been proposed that p38α/β promote adipogenesis through the phosphorylation of C/EBPβ [179], a key transcription factor for adipose tissue differentiation [180]. Experiments with p38 inhibitors and p38α knockout cells have shown that p38 phosphorylates and activates C/EBPβ, leading to adipogenesis via PPARγ upregulation [181]. However, lack of p38α in preadipocytes did not affect their differentiation to adipocytes, nor did it affect changes in the differentiation markers evaluated in the major fat depots [177]. 

Therefore, specific members of p38 family differentially regulate adipose tissue metabolism.

#### 2.3.3. The Role of p38s in Pancreatic β-Cells

Several reports suggest that activation of the p38s pathway promotes β-cell apoptosis and that p38s activity is increased in pancreatic islets of T2D model mice [182]. Indeed, p38 inhibitor SB203580, which targets p38α and p38β, but not other members of the family, can lower blood glucose by improving β-cell function mediated through a reduction in β-cell apoptosis [183]. Additionally, the inhibition of p38 (both p38α and p38β) or JNKs activity increased paired box 6 (Pax6) levels in high glucose-treated β-cells. Pax6 is known to be a key transcription factor playing pivotal roles in β-cell function, including cell survival, insulin biosynthesis, and secretion [184]. Mechanistically, calcium-independent phospholipase A2β (iPLA2β), downstream target of p38, seems to be involved in islet β-cell apoptosis [185]. Moreover, ER stress-induced ASK1/p38 activation similarly induced pancreatic β-cell death [182]. However, this involvement of p38α/β does not seem to play a key role in apoptosis [65]. It has also been shown that activated p38α/β MAPK probably inhibits the ERK pathway [65].

Different studies have suggested that p38 isoforms may present various functions for β-cells. For instance, p38δ controls insulin secretion through the phosphorylation and inhibition of PKD1 [186]. In the absence of p38δ, activity of PKD1 is enhanced, which promotes trans-Golgi network function dynamics and consequently stimulates insulin secretion. Consistently, mice lacking p38δ present elevated insulin levels and are protected against diabetes. Interestingly, stress-induced β-cell death is also prevented by the deletion of p38δ [186]. Of note, p38δ–PKD1 axis also regulates autophagy and lysosomal degradation of insulin nascent granules in β-cells, which allow appropriate response to the nutrient availability and maintain the secretory function of β-cells [187]. However, the exact molecular mechanisms by which the p38δ–PDK1 pathway is regulated remain to be defined. Furthermore, p38α directly affects the glucose uptake in pancreatic β-cells through TSH signaling by up-regulating the expression of GLUT2 gene [188]. Finally, p38γ may have an important role in TNFα-mediated downregulation of ATP-binding cassette transporter A1 (ABCA1) expression, leading to suppression of insulin secretion [189]. 

To sum up, distinct p38 members promote pancreatic β-cell death and suppress insulin secretion.

#### 2.3.4. p38s Define Inflammatory Response to Control Metabolism

The p38 MAPK family regulates several cytokines levels and therefore controls systemic inflammation. Inhibition of p38, either by inhibitors (targeting p38α and p38β) or negative dominance, leads to reduced IFN-γ production by T helper Th1 cells but does not affect IL-4 production by Th2 cells [190,191]. Suppression of the p38 pathway significantly suppressed the release of TNFα and prostaglandin E2 (PGE2) from macrophages and inhibited cyclooxygenase-2 (COX-2) expression [192]. Macrophage deletion of p38α impairs the innate immune response, indicating that p38α activation is important for cytokine production (TNFα, IL-12, and IL-18), as well as for the activation of transcription factors C/EBP-β and CREB [193]. In parallel, myeloid-specific p38γ and p38δ gene ablation impaired cytokine production of TNFα, IL-1β, and IL-10 by blocking ERK1/2 protein kinase pathway activation in macrophages and in dendritic cells [194]. Translational elongation of nascent pro-TNFα protein is mediated by eukaryotic elongation factor 2 (eEF2) kinase, which is inhibited by p38γ/p38δ-mediated phosphorylation [195]. The lack of p38γ and p38δ in myeloid cells also impaired the migration of neutrophils to the liver and thus protected against steatosis and further hepatic damage [155]. The low adhesion and higher rolling velocity observed in neutrophils lacking p38γ and p38δ were strongly similar to previous reports using global and myeloid-specific deletion of p38δ in mice [196]. Neutrophils have been shown to be important mediators of alcoholic fatty liver disease [197], and p38δ is known to regulate the neutrophil inflammatory response in the lungs by controlling PKD1 activity [196].

Therefore, p38 members might control metabolism also by regulation of immune responses.

#### 2.3.5. p38s in Regulation of Skeletal Muscle Function

Increased activation of p38 MAPK has been observed in skeletal muscle from T2D patients [198]. Indeed, p38 activation was found to be increased in the diabetic myotubes, and p38α/β kinases were identified as a key regulator of the expression of different proinflammatory genes associated with an upregulated gene sets in diabetic myotubes [199]. However, p38α/β inhibition in diabetic skeletal muscle cells did not improve the retained defect of insulin-stimulated glucose uptake despite decreased inflammatory cytokine expression [199]. Thus, increased infiltration of proinflammatory macrophages could contribute to the reduced myoblast differentiation, associated with obesity-induced muscle loss by secreting the inflammatory cytokine TNFα via p38 signaling pathway [200]. As previously mentioned, increased infiltration of proinflammatory macrophages in skeletal muscles is noted in obesity and is associated with muscle insulin resistance. Importantly, cachectic cancer cells have been found to secrete many inflammatory factors that have rapidly led to high levels of FA metabolism and to the activation of a p38 stress-response signature in skeletal muscles, before the manifestation of cachectic muscle atrophy occurred [201]. These secreted factors rapidly induce excessive FA oxidation in human myotubes, which leads to oxidative stress, p38 activation, and impaired muscle growth. Moreover, FA binding protein 4 (FABP4) inhibitor reduces FA-induced ER stress-associated inflammation in skeletal muscles by reducing p38 MAPK activation [202]. Additionally, angiotensin II induces insulin resistance in skeletal muscle by a mechanism dependent on oxidative stress and activation of p38, which results in decreased function of glucose transporter type 4 [203]. On the other hand, the deletion of MKP-1, which results in the activation of p38s and JNKs, leads to increased oxidative metabolism in muscles and elevated energy expenditure in mice and thus prevents obesity and insulin resistance [204].

Several reports suggest that p38 isoforms could present different metabolic functions in skeletal muscle cells. The α isoform positively regulates muscle atrophy [205], whereas the γ isoform regulates endurance exercise-induced mitochondrial biogenesis and angiogenesis [206] and glucose uptake [207]. p38γ is also involved in muscle-specific exercise-induced skeletal muscle adaptation, and it seems to be required for the upregulation of PGC-1α in mitochondrial biogenesis and angiogenesis in response to exercise and nerve stimulation in mice. In addition, p38γ improves basal glucose uptake and lowers contraction-stimulated glucose uptake, partially by affecting levels of glucose transporter 4 expression in skeletal muscle [207]. On the contrary, the β isoform appears to be responsible for the catabolic effect of p38 MAPK because it activates the transcription factor C/EBPβ that up-regulates atrogin1 [208]. Indeed, activation of type IIB activin receptor (ActRIIB) by activin A induces muscle catabolism, primarily through the activation of p38β-mediated catabolic signaling that activates ubiquitin-proteasome and autophagy-lysosome pathways [209]. 

To sum up, members of p38 family play a central role in the regulation of skeletal muscle metabolism and are implicated in the development of metabolic diseases.

#### 2.3.6. p38s in Central Regulation of Metabolic Homeostasis

Similarly to ERK1/2, fasting activates p38s in the ARC and paraventricular nucleus in mice [86,87], and this activation is reversed by refeeding [87]. Fasting also regulates *Fgf21* production in tanacytes via p38α/β kinase [210]. Tanycytes, the hypothalamic ependymo-glia, sense FFA to maintain body lipid homeostasis through Fgf21 signaling within the hypothalamus. In fact, tanycytes store palmitate in lipid droplets and oxidize it, leading to the activation of a ROS/p38 signaling pathway, which is essential for tanycytic Fgf21 production upon palmitate exposure. In parallel, in diabetic untreated rats presenting hyperglycemia, p38s activity showed significant alterations in striatum, hippocampus, hypothalamus and pons medulla that correlated with the changes of levels of both catecholamines, dopamine, and epinephrine [211]. These changes were reversed in insulin-treated diabetic rats, suggesting that p38s may regulate the rate of either the synthesis or release of dopamine and epinephrine in the corresponding brain areas. Moreover, p38s seems also to be implicated in different signaling pathways regulating insulin-sensitive tissues within the hypothalamus. Nuclear factor of activated T cells 3 (NFATc3)-knockout markedly attenuated HFD-triggered insulin resistance, liver steatosis, and importantly neuroinflammation and apoptosis, which was attributed to the reduced activation of p38s/JNKs signaling pathways [212]. Interestingly, p38s or JNKs activation could rescue inflammatory response and apoptosis in NFATc3-KO astrocytes. In the same context, resistin, a polypeptide secreted by adipose tissue in rodents and by macrophages in human, promoted the activation of p38s and JNKs, enhanced serine phosphorylation of IRS1, and increased the expression of the pro-inflammatory cytokine IL-6 in the hypothalamus and key peripheral insulin-sensitive tissues [213]. 

To sum up, p38 activation in the hypothalamus determines peripheral metabolism.

#### 2.3.7. Summary

**Figure 3:**
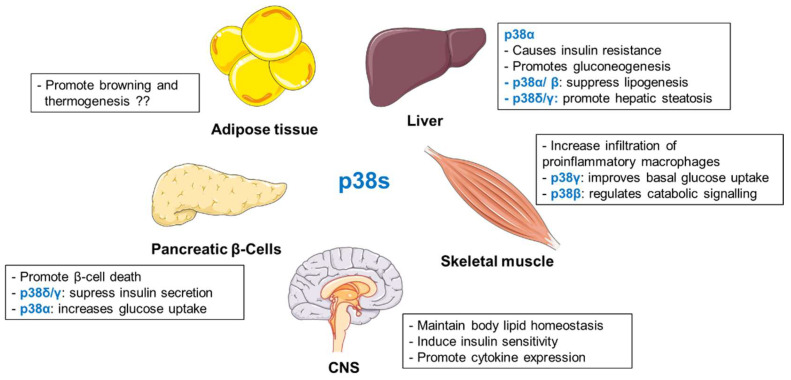
Organ-specific roles of p38 isoforms in regulation of metabolism.

Several studies place p38s as important regulators of metabolic homeostasis (Figure 3). Mice lacking hepatic p38α exhibited reduced fasting glucose levels and impaired gluconeogenesis [154]. p38α/β were also shown to be required for glucagon and fasting-mediated suppression of hepatic lipogenesis [166]. Interestingly, p38γ and p38δ are responsible for the development of liver steatosis [155]. In adipocytes, specific members of p38 family differentially regulate adipose tissue metabolism [173,176,177]. Findings from different laboratories present an ambiguous picture of the role of p38α in browning and thermogenesis [173,176,177]. Similar to adipocytes, p38 isoforms present various functions in pancreatic β-cells and skeletal muscle [186,187,188,189,207,208]. In pancreatic β-cells, p38δ/γ suppress insulin secretion [186,187,189], whereas p38α promotes glucose uptake [188]. In skeletal muscle cells, p38γ stimulates glucose uptake [207]. On the contrary, the β isoform appears to be responsible for the catabolic effect [208]. Finally, p38 activation in the hypothalamus seems to maintain body lipid homeostasis [210] and to regulate insulin-sensitivity in peripheral tissues as well as pro-inflammatory cytokine expression [212,213]. 

### 2.4. ERK5 Kinase

ERK5, also termed big mitogen-activated protein kinase-1 (BMK1), is the most recently identified member of the conventional MAPK family [214]. ERK5 is activated by different extracellular stimuli, including growth factors, inflammatory cytokines, oxidative and osmotic stresses, ischaemia, and hypoxia. The MAPKKKs activated by these extracellular stimuli are MEKK2 and MEKK3, which specifically phosphorylate the MAPKK MEK5, which in turn, directly activates ERK5 through phosphorylation of both tyrosine and threonine residues of the Thr-Glu-Tyr motif present within the activation loop of the ERK5 kinase domain. Because of its unique extended C-terminus containing a nuclear localization signal, two proline-rich regions, and a transcriptional activation domain, it appears that the most significant role of ERK5 is to regulate a number of downstream transcription factors. A variety of downstream substrates, including other kinases such as serum- and glucocorticoid-induced protein kinase (SGK) and transcription factors such as MEF2, Elk-1 and Sap1a are phosphorylated by ERK5 [214] (Table 1). Although it is associated with a diverse range of cellular processes including cellular proliferation, migration, survival, and angiogenesis, little is known regarding the importance of ERK5 in metabolism regulation and energy homeostasis (reviewed in [1,2,214]).

Systemic knockout of ERK5 was shown to be embryonically lethal in mice because of defects in angiogenesis and vascular formation [215]. No obvious phenotype has been reported from hepatocyte-specific ERK5 knockout mice [216]. However, ERK5 deficiency in LepR-expressing neurons resulted in an obesity phenotype with enhanced WAT mass due to increased adipocyte size, but only in female mice fed a normal chow diet [217]. Moreover, impaired glucose homeostasis along with decreased physical activity, energy intake, and energy expenditure was observed in these animals. Although it has been reported that the deletion of ERK5 in adipose tissues using Adiponectin-Cre increased adiposity due to increased food intake, dysregulated secretion of adipokines, leptin resistance, and impaired glucose handling were also found in these mice [218]. In parallel, ERK5 was found to protect against pancreatic β-cell apoptosis and hyperglycaemia in mice by interrupting the ER stress-mediated apoptotic pathway [219]. Suppression of ERK5 activity in pregnant mice significantly decreased β-cell proliferation without affecting β-cell apoptosis, resulting in increased random blood glucose levels and impaired glucose response in mice [220]. ERK5 seemed to activate cyclin D1 to promote gestational β-cell proliferation. 

#### Summary

**Figure 4:**
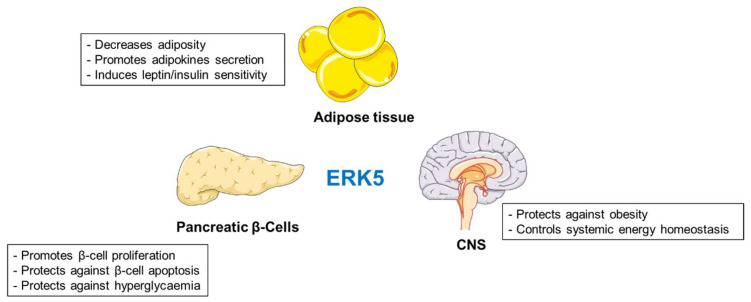
The impact of ERK5 on various tissues and organs.

More efforts are needed to elucidate the specific roles of ERK5 in regulation of metabolism. However, activation of ERK5 in multiple tissues seems to protect against development of obesity and diabetes (Figure 4).

## 3. The Atypical MAPKs

Atypical MAP kinases include ERK3/ERK4, NLK, and ERK7. Much less is known about their regulation, substrate specificity, and physiological functions. Atypical MAPKs are not organized into classical three-tiered kinase cascades. In addition, the Thr-X-Tyr motif is absent in ERK3/4 and NLK, where a Gly or Glu residue replaces the Tyr. ERK7 contains the motif Thr-Glu-Tyr in its activation loop, but phosphorylation of these residues appears to be catalyzed by ERK7 itself, rather than by an upstream MAPKK (reviewed in [1,2]). While the conventional MAPKs signaling pathways that underlay metabolism have been well-studied, regulation of metabolism via atypical MAPKs signaling pathways is poorly understood.

Whereas the biological role of ERK4 is currently unknown, ERK3 has been shown to be involved in a number of biological functions, including cell proliferation, cell cycle progression, and cell differentiation [2]. ERK3 is a highly unstable protein that is constitutively degraded by the ubiquitin-proteasome pathway in proliferating cells [221]. In the cytoplasm, ERK3 is activated by the conventional PKCβ [222] and targets downstream activation of MAPK-activated protein kinase-5 (MK5) [223,224]. High throughput screen for kinases regulating lipolysis in adipocytes revealed ERK3 as a major factor promoting adipocyte function. Consistently, targeted deletion of ERK3 in mouse adipocytes inhibits lipolysis, but elevates energy dissipation, promoting lean phenotype and ameliorating diabetes [225]. Mechanistically, β-adrenergic stimulation stabilizes ERK3, leading to the formation of a complex with the cofactor MK5, and thereby driving lipolysis. ERK3/MK5 pathway promotes lipolysis by promoting the expression of a major lipase, adipose triglyceride lipase (ATGL), in a FOXO1-dependent manner [225]. On the other hand, depletion of ERK3 in adipocytes promotes UCP1 expression and energy dissipation by brown, as well as white, adipose tissue, thereby protecting against obesity and diabetes [225]. Pregnancy induces peripheral insulin resistance, which is normally compensated by increased β-cell proliferation, expansion of islet volume, and increased insulin synthesis and secretion. In fact, ERK3 is upregulated during pregnancy [226]. ERK3 expression is modulated by prolactin (PRL) in isolated rat pancreatic islets and seems to be involved in glucose-induced insulin release. This mechanism involves activation of the conventional protein kinase C (PKC), leading to ERK3 serine phosphorylation and association with microtubule-associated protein 2 (MAP2) [226]. ERK3 seems also to play a key role in IL-8 production in a kinase-independent manner through its interaction with c-Jun. ERK3 controls the DNA-binding activity of the activating protein 1 (AP-1) transcription factor, which is critically required for the activation of several cytokines, including IL-8, by regulating c-Jun nuclear abundance [227]. These data indicate that ERK3 plays a central role in the regulation of adipocyte function. However, its function in other tissues needs to be explored.

NLK is part of the non-canonical Wnt signaling pathway that promotes osteogenesis and inhibits adipogenesis of mesenchymal stem cells [228,229,230]. In addition, NLK plays an important role in maintaining pre-adipocytes in an undifferentiated state by inhibiting adipogenic gene expression [231,232]. It has been reported that NLK-deficient mice exhibit increased adipocyte numbers in the bone marrow [233]. Moreover, the polymorphism of the NLK gene was significantly associated with human fat body mass [232], and with intramuscular fat content and FA composition traits in pigs [234].

Despite the lack of identified substrates, ERK7 and its human ortholog ERK8 play important biological functions, notably in the regulation of cell proliferation and in the response to estrogens and glucocorticoids [2]. In mammals, insulin-like signaling is mediated by insulin and insulin-like growth factors through their respective receptors. Drosophila possesses a single insulin-like receptor, which is activated by insulin-like peptides (dILPs), mainly expressed and secreted by a group of median neurosecretory cells, also known as insulin-producing cells (IPCs). Interestingly, ERK7 is essential to inhibit dILP secretion upon impaired ribosome biogenesis, and it acts epistatically to p53 [235]. Both p53 and ERK7 activities within the IPCs contribute to the regulation of dILP secretion in response to nutrient status. The p53- and ERK7-dependent ribosome surveillance pathway seems to serve as a local branch of the IPC-regulating nutrient-sensing network, parallel to the humoral signals derived from the fat body. 

### Summary

**Figure 5:**
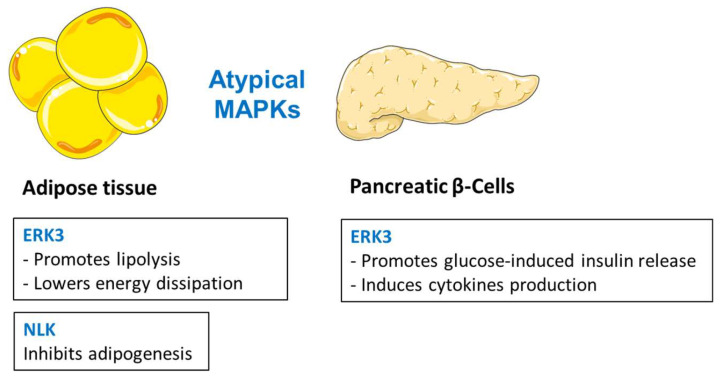
The role of atypical MAPKs in regulation of metabolism.

The role of atypical MAPK in the regulation of metabolism is relatively poorly explored. However, ERK3 and NLK emerge as important regulators of adipocyte and pancreatic β-cell function (Figure 5). ERK3 promotes lipolysis in adipose tissue and suppresses energy dissipation in this organ [225]. ERK3 also promotes insulin secretion from the pancreatic β cell [226]. NLK regulates adipose tissue homeostasis by suppressing adipogenesis [231,232]. 

## 4. Conclusions, Future Perspective and Therapeutic Implications

In summary, this review provides updated insights into the critical functions of MAPK-signaling pathways and their role in the development of metabolic diseases such as obesity and T2D. While initially research into MAPKs and metabolism has focused on the established ERK1/2, JNKs, and p38s MAPK families, it is now becoming apparent that ERK5, the most recently discovered MAPK, and atypical MAPKs, mainly ERK3, also play an important role in the regulation of metabolic homeostasis. Moreover, as described above, different studies suggest that the distinct isoforms of ERK1/2, JNKs, and p38s impact metabolism in diverse manners. However, further studies are needed to fully clarify the role of each of the members of the MAPK family in the development of metabolic disorders. Additionally, ERK3, ERK4, ERK5, ERK7/8, and NLK have not been thoroughly studied in vivo yet, and a limited number of substrates and functions have been annotated to these kinases. The use of genetically modified animals, especially conditionally deficient mouse models and molecular targeting strategies able to specifically inhibit particular MAPKs signaling pathways enabled to uncover the specific functions of the kinases from this family in organs implicated in regulating metabolic homeostasis (Table 2). However, further studies are needed to fully define the physiological roles of kinases from MAPK family in the regulation of metabolism and the development of metabolic diseases.

Importantly, understanding the regulation mechanisms of signal transduction by conventional MAPKs has provided useful information for the development of specific inhibitors, which eventually could be used in the clinical practice. In fact, drugs targeting MAPK pathways have already provided striking clinical responses, especially in inflammatory diseases and cancers [236,237]. Thus, targeting these kinases might also represent a promising avenue for the treatment of metabolic disorders such as obesity, diabetes, and atherosclerosis. Unfortunately, to date, conventional MAPKs are used mainly as markers for metabolic research trials (registered in clinicaltrial.gov: NCT02498119, NCT00330967, NCT03811717, NCT00524901, NCT01272674, NCT02498119, NCT00291902, NCT02367287, NCT02367287), and there is a very limited number of research trials targeting these kinases directly for treatment of obesity and diabetes. The dual αβ subtype p38 inhibitor, losmapimod, modestly reduced vascular inflammation in stable atherosclerosis subjects, as measured by 18FDG-PET/CT [238]. Moreover, a significant reduction of inflammation in visceral fat and a persistent reduction in high sensitivity C-reactive peptide (hsCRP) were observed. However, BMS-582949, a novel highly selective p38α inhibitor, does not lead to a significant reduction of either circulating inflammatory biomarkers or imaging measures of atherosclerotic inflammation [239]. These results indicate that inhibition of p38β might be required in order to obtain the desired anti-inflammatory effects in atherosclerosis. Overall, currently available data suggest that inhibition of specific members of conventional and/or atypical MAPKs might be beneficial for the treatment of metabolic diseases. However, since MAPKs play a central role in normal biological functions, direct inhibition of these kinases may also have unexpected consequences. The development of highly selective inhibitors is therefore essential for the safe therapy of metabolic disorders.

## Figures and Tables

**Table 1:** Conventional MAPKs signaling cascades and their major substrates.

Stimulus	MAPKKK	MAPKK	MAPK	Substrates and Biological Functions
Growth factors, ligand for GPCRs, cytokines, osmotic stress, microtubule disorganization, and insulin.	RAF family (ARAF, BRAF, CRAF)	MEK 1/2	**ERK1/2**	RSK family (gene transcription, cell proliferation, growth, and survival) MSK1/2 (gene transcription, nucleosome dynamics) MNK1/2 (mRNA translation) Elk-1 (transcription of c-Fos) c-Fos (transcription) Synapsin I, focal adhesion kinase [FAK] and myosin light-chain kinase (actin polymerization) Neurofilaments and paxillin (cytoskeleton organization) CD120a, spleen tyrosine kinase [SYK], and calnexin (plasma membrane dynamics) Death-associated protein kinase [DAPK] (cell death) Tuberous sclerosis complex 2 [TSC2] (nutrient sensing) Nuclear factor of activated T-cells [NF-AT], myocyte enhancer factor 2 [MEF2] and c-Myc (transcription) Signal transducer and activator of transcription 3 [STAT3] (signaling)
Stress (hypoxia, UV, and ionizing radiation), cytokines, growth factors, pathogens, toxins, drugs, metabolic changes (obesity and hyperlipidaemia).	MEKK1 to –4 Mixed lineage kinase 1/2/3 [MLK1/2/3] Tumor progression locus 2 [Tpl-2] Delta-like non-canonical Notch ligand [DLK] TAO1/2 TGF-β-activated kinase 1 [TAK1] Apoptosis signal-regulating kinase 1/2 [ASK1/2]	MKK4 MKK7	**JNKs**	c-jun, (transcription, cell cycle and apoptosis) BH3-only family of Bcl2 proteins (apoptosis) p53 (apoptosis) Activating transcription factor 2 [ATF-2], nuclear factor of activated T-cells, cytoplasmic 1 [NF-ATc1], Elk-1, Heat shock factor protein 1 [HSF-1], STAT3, c-Myc, JunB (transcription)
Oxidative stress, UV irradiation, hypoxia, ischemia, inflammatory cytokines, ligand for GPCRs, and Rho family GTPases.	MEKK1 to -3 MLK2/3 ASK1 Tpl-2 TAK1 TAO1/2	MKK3 MKK6 TAK1 binding protein 1 [TAB1] ZAP70 LCK	**p38s**	MSK1/2 (gene transcription, nucleosome response) MNK1/2 (mRNA translation) p53 (preventing p53 proteasomal degradation) ATF (regulation of ER stress response) ATF-2 and Nuclear factor NF-kappa-B [NF-κB] (expression of inflammatory cytokines) Elk-1 (transcription) protein kinase D1 [PKD1] (Trans-Golgi dynamics, signaling)
Growth factors, inflammatory cytokines, oxidative and osmotic stresses, ischaemia, and hypoxia.	MEKK2 MEKK3	MEK5	**ERK5**	SGK (kinase) MEF2, Elk-1 and Sap1a (transcription)

**Table 2:** MAPKs and metabolic phenotype observed in genetic in vivo experiments. (–) indicates negatively regulated. (+) indicates positively regulated. ?? indicates parameter was not investigated.

	Liver	Adipose Tissue	Pancreatic β-Cells	Skeletal Muscle	CNS
**ERK1/2**	(-) insulin sensitivity	(-) insulin sensitivity (+) adiposity	(+) glucose-stimulated insulin secretion (+) β-cell survival	??	(+) energy expenditure (-) adiposity (-) food intake
**JNKs**	contradictory results	(-) hepatic insulin sensitivity (+) hepatic steatosis	β-cell dysfunction	no effect on adiposity contradictory results on insulin sensitivity	(+) adiposity (-) glucose tolerance (-) insulin sensitivity (+) hepatic steatosis
**p38s**	**p38α**: (+) luconeogenesis (+) fasting hyperglycemia **p38α/β** (-) lipogenesis **p38γ/p38δ** (+) hepatic steatosis	Contradictory results	**p38δ**: (-) insulin secretion **p38α**: (+) glucose uptake	**p38γ**: (+) glucose uptake	??
**ERK5**	no obvious phenotype	(-) adiposity (+) leptin/insulin sensitivity	(-) hyperglycaemia	??	(-) adiposity
**ERK3**	??	(-) insulin sensitivity (+) adiposity	??	??	??
